# Could Glyphosate and Glyphosate-Based Herbicides Be Associated With Increased Thyroid Diseases Worldwide?

**DOI:** 10.3389/fendo.2021.627167

**Published:** 2021-03-19

**Authors:** Renata Marino Romano, Jeane Maria de Oliveira, Viviane Matoso de Oliveira, Isabela Medeiros de Oliveira, Yohandra Reyes Torres, Paula Bargi-Souza, Anderson Joel Martino Andrade, Marco Aurelio Romano

**Affiliations:** ^1^ Department of Medicine, State University of Central-West, Guarapuava, Brazil; ^2^ Instituto Federal do Paraná, Irati, Brazil; ^3^ Instituto para Pesquisa do Câncer, Guarapuava, Brazil; ^4^ Department of Chemistry, State University of Central-West, Guarapuava, Brazil; ^5^ Department of Physiology and Biophysics, Institute of Biological Sciences, Federal University of Minas Gerais, Belo Horizonte, Brazil; ^6^ Department of Physiology, Federal University of Paraná (UFPR), Curitiba, Brazil

**Keywords:** thyroid diseases (source: MeSH NLM), glyphosate (N-[phosphonomethyl]glycine), glyphosate-based herbicides, thyroid gland—anatomy and histology, allostasis, reactive oxygen species, endocrine-disrupting chemicals

## Abstract

The increased incidence of thyroid diseases raises a series of questions about what the main predisposing factors are nowadays. If dietary restriction of iodine was once a major global health concern, today, the processes of industrialization of food and high exposure to a wide variety of environmental chemicals may be affecting, directly or indirectly, thyroid function. The homeostasis of hypothalamus–pituitary–thyroid (HPT) axis is finely regulated through the negative feedback mechanism exerted by thyroid hormones. Allostatic mechanisms are triggered to adjust the physiology of HPT axis in chronic conditions. Glyphosate and glyphosate-based herbicides are pesticides with controversial endocrine disrupting activities and only few studies have approached their effects on HPT axis and thyroid function. However, glyphosate has an electrophilic and nucleophilic zwitterion chemical structure that may affect the mechanisms involved in iodide oxidation and organification, as well as the oxidative phosphorylation in the ATP synthesis. Thus, in this review, we aimed to: (1) discuss the critical points in the regulation of HPT axis and thyroid hormones levels balance, which may be susceptible to the toxic action of glyphosate and glyphosate-based herbicides, correlating the molecular mechanisms involved in glyphosate toxicity described in the literature that may, directly or indirectly, be associated to the higher incidence of thyroid diseases; and (2) present the literature regarding glyphosate toxicity in HPT axis.

## Introduction

Thyroid diseases are the second leading cause of endocrine disorders (1). In iodine-sufficient regions, autoimmune thyroiditis is the main cause of thyroid dysfunction (2). The most common are Hashimoto’s disease (also known as chronic autoimmune thyroiditis or autoimmune hypothyroidism) and Graves’ disease (or autoimmune hyperthyroidism) (3, 4). Besides iodine deficiency or excess, genetic predisposition, selenium deficiency, infectious agents (such as *Yersinia enterocolitica*), and syndromic conditions such as Down syndrome and Turner syndrome are associated with autoimmune thyroiditis (2). The incidence of thyroid cancer is increasing worldwide. Age-standardized incidence rate from 1990 to 2013 showed an increase of 20%. In some high-income countries, the increase is partially attributed to better screening programs. However, the causes may be considered multifactorial with geographic influences, iodine supplementation, environmental factors, such as endocrine disruptors and carcinogens (5).

Glyphosate is a very effective, non-selective and post-emergent herbicide used to control harmful weeds for several crops (6, 7). It represents about 65% of the total herbicides used in Brazil (8). Glyphosate has been detected in urine samples of individuals occupationally, para-occupationally, or environmentally exposed to the herbicide, in urban and rural areas (9), raising the concerns of the toxic effects in children, pregnant women, basically in all age groups. Glyphosate has been associated with several diseases, including thyroid disorders (10). The increase in the use of glyphosate along the years presents a positive correlation with the increase in the prevalence of several intestinal diseases, such as celiac disease (10). Celiac disease and thyroid disorders prevalently coexist (11) and it is speculated that there is a possible link between exposure to glyphosate and the development of these pathologies (10). Recently, glyphosate was associated with an increased risk for thyroid diseases in farmers (12, 13). However, the regulatory agencies did not find any evidence to classify glyphosate as an endocrine-disrupting chemical or a carcinogenic agent (7, 14). Despite this, there are evidences between glyphosate exposure and incidence of Non-Hodgkin lymphoma (15–17) and the International Agency for Research on Cancer (IARC/WHO) classified it as “probably carcinogenic to humans” (Group 2A) (9, 18–22). This discrepancy is possibly due to the evaluation of glyphosate alone by EFSA and ATSDR, not considering the chemical agents contained in lesser amounts in commercial formulations.

In this context, the objectives of this review are discuss the critical points in the regulation of HPT axis and thyroid hormones levels balance, which may be susceptible to the toxic action of glyphosate and glyphosate-based herbicides, correlating the molecular mechanisms involved in glyphosate toxicity described in the literature that may, directly or indirectly, be associated to the higher incidence of thyroid diseases.

## The Maintenance of Hypothalamus–Pituitary–Thyroid Axis Homeostasis and its Allostasis-Adaptive Responses

### Physiology of the Hypothalamic–Pituitary–Thyroid Axis

**Figure 1:**
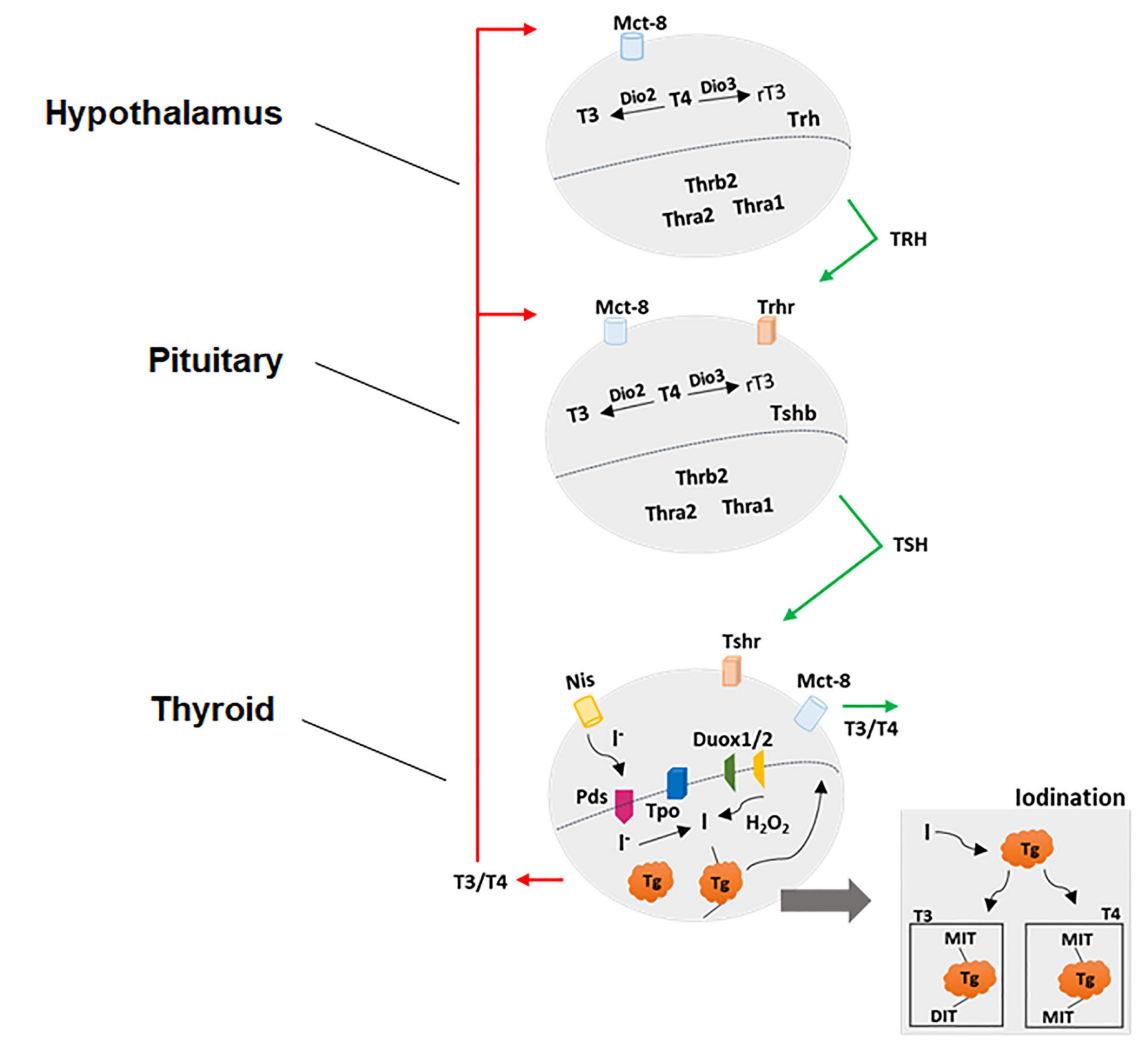
Physiological control of hypothalamic-pituitary-thyroid axis. See the text for detailed explanations.

The neuroendocrine system, which comprises the hypothalamus–pituitary-gland axis, is responsible for the homeostatic regulation of most physiological processes such as reproduction, growth, metabolism, energy balance and response to stress. In particular, the HPT axis controls the thyroid gland metabolism and synthesis and the secretion of thyroid hormones (THs) (**Figure 1**). The neurons from hypothalamic paraventricular nucleus (PVN) secrete the tripeptide Thyrotrophin-Releasing Hormone (TRH) in the hypothalamic-pituitary portal system, reaching the anterior pituitary gland. The TRH interacts with its receptor (TRH-R) located on plasma membrane of thyrotrophs and lactotrophs and stimulates Prolactin (PRL) and Thyroid-Stimulating hormone (TSH) biosynthesis and secretion (23, 24). After being released into the bloodstream, the TSH reaches its receptor (TSH-R) in the basolateral plasma membrane of thyroid follicular cells and stimulates the thyroid gland metabolism and several stages involved in the biosynthesis and secretion of THs (25, 26). In humans, the thyroid gland synthesizes and secretes about 75% of 3,5,3’,5’-tetraiodothyronine (T4 or thyroxine) and 25% of 3,5,3’- triiodothyronine (T3) under regulation of hypothalamus and pituitary gland (27, 28).

Once it is in the bloodstream, the highest percentage of THs (about 95–99%) is carried by specific binding proteins such as Thyroid hormones binding proteins (TBGs) and Transthyretin or by albumin in dynamic equilibrium with the free fraction (about only 1–5%). The free fraction is responsible for the actions in the target tissues, which include virtually all cells. The proper functioning of the molecular mechanisms involved in actions of THs, including the regulation of HPT axis by negative feedback, depends on several cellular components as specific transporters and signals mediated by enzymes and receptors. Firstly, the transport of THs through the plasma membrane is mediated by the Monocarboxylate Transporters 8 and 10 (MCT8 and MCT10) and by Solute Carrier Organic Anion Transporter family member 1C1 (OATP1C1), however; only MCT8 has been identified as being specific to transport THs (29–31). Secondly, the most abundant product of thyroid is the T4, while about 90% of T3 in bloodstream is a product of intracellular conversion of T4 to T3 by a family of selenocysteine-containing enzymes in peripheral tissues. These enzymes are named as 5’-deiodinases and catalyze the removal of one iodine from the thyroid products generating intermediate thyroid products, which in turn exert a wide range of genomic and non-genomic actions in several tissues (32). Thirdly, the transcriptional or genomic actions of THs are mainly mediated by Thyroid Hormone Receptors Alpha (THRA) and Beta (THRB); transcription factors that belong to the nuclear receptor superfamily and modulate the transcriptional rate of target genes. THRs are encoded by two different genes, Thra and Thrb, however, several isoforms are generated by alternative splicing (33). The THRs exhibit higher affinity with T3 (compared to T4 and other THs such as T2 and reverse T3–rT3) and are associated with specific sequences in DNA called thyroid hormone-responsive elements (TREs), located in the regulatory regions of a wide variety of target genes. Briefly, the THR-T3 interaction results in the recruitment of several co-activating proteins and positive modulation of the transcription of target genes (34). Finally, non-genomic actions triggered by THs were described taking place in the plasma membrane in association with the arginyl-glycyl-aspartic acid domain (RGD) present in αVβ3 integrin, in binding sites of rough endoplasmic reticulum (RER) and mitochondria, as well as through the association of THRs with specific enzymes in cytosol, such as PI3K. Among others, these actions include the modulation of glucose uptake and ions transport through the plasma membrane, alterations in cytoskeleton dynamics, kinase activities and transcripts stability (35).

### The Allostasis-Adaptive Responses of HPT Axis

The HPT axis feedback control is a dynamic process which exhibits an important adaptive response, basically ensured by two mechanisms: relaying and conversion. The control of TSH secretion by TRH and of T4 by TSH are examples of relaying. On the other hand, the central and peripheral T4 conversion to T3 by deiodinases and the transport of THs by plasma and OATP1C1 and MCT8 transporters exemplify the conversion mechanism (36). Thus, alterations in the activity or expression of deiodinases and thyroid hormones transporters are associated to the adaptive response observed in several stressful conditions in an attempt to maintain the required TH concentration in serum. In this sense, the DIO2 activity in tanycytes maintains constant in physiological conditions. During an infectious condition, however, an increase in DIO2 activity leads to higher levels of hypothalamic T3, which might be the molecular mechanism associated to the central hypothyroidism observed in the nonthyroidal illness syndrome (37).

These reactive regulations of HPT axis are characterized as thyroid allostasis-adaptive responses types 1 and 2 (38). The type 1 allostasis is usually characterized by a low or normal TSH level and free T4 levels, low total T4, T3 and free T3 serum concentrations and higher levels of rT3. It is mostly commonly observed in hospitalized patients presenting non-thyroidal illness syndrome (NTIS) or thyroid allostasis in critical illnesses, tumors, uremia, and starvation (TACITUS). In hospitalized patients, the NTIS or TACITUS is commonly observed (38). The type 2 allostasis is usually observed in psychosocial stress, pregnancy, obesity associated with metabolic syndrome, adaptation to cold and post-traumatic stress disease. In contrast to type 1 allostasis, the TSH and free T4 levels are high or normal, the free T3 and total T4 and T3 levels are high, while the levels of rT3 are low. These alterations are associated with increased levels of THs binding protein levels, upregulation of DIO1 and 2 activities and downregulation of DIO3.

Therefore, there are substantial differences regarding the molecular mechanisms involved in types 1 and 2 adaptive allostatic-responses of HPT axis. In general, they are the result of an HPT axis adaptation that is acutely essential to adjust the thyroid and body metabolism in critical illnesses and several stressful conditions. However, the exposure to chronic stressful conditions, drugs or endocrine disruptors can overcome the HPT axis adaptive capacity determined by the saturation of receptors and enzymes, leading to allostatic overload that may culminate in pathological thyroid disorders. Rats prepubertally exposed to acrylamide (AA) present several alterations in the HPT axis with higher levels of THs in serum. Interestingly, compensatory mechanisms were observed in TH-target tissues, such as an increase in Dio3 mRNA expression in the liver and a reduction in Mct8 transcript content in the hearts of AA-treated rats, suggesting an allostatic regulation (39). Similar mechanisms of allostatic regulation were evidenced in the HPT axis and target-tissues of rats prepubertally exposed to silver nanoparticles (40).

Albeit the allostasis-adaptive responses of HPT axis are essential for the proper adjust to critical conditions, they difficult the clinical diagnosis of thyroid diseases, since the hormonal levels that comprise this axis exhibit a wide range of reference values. Furthermore, many patients are classified in the subclinical hypothyroidism or hyperthyroidism category and remain without treatment until the rise of these respective thyroid pathological conditions, hypothyroidism and hyperthyroidism (41).

Environmental stressors such as exposure to endocrine-disrupting chemicals that target the HPT axis may impose an additional allostatic burden (38). This is of particular concern during fetal and early postnatal life given the critical role THs exert on developmental processes, including body growth and neurodevelopment, and the lifelong consequences that may result from improper TH action on developing organisms, including cognitive deficits, impairment of psychomotor and language abilities, and other behavioral problems (42). Although thyroid hormone status during fetal life resembles a type 1 allostatic response with low circulating total and free T3, the levels of T3 in the brain are preserved due to an increased DIO2 activity (38). Thus, chemical exposures that alter the fine hormonal balance during this critical developmental stage may disrupt TH signaling and result in adverse neurodevelopmental outcomes.

## Thyroid Hormones Synthesis Pathway as a Possible Target to Toxicants

DUOX1 and DUOX2 are glycoprotein members of the NADPH oxidase family that generate hydrogen peroxide (H2O2). These proteins form a heterodimer with the specific maturation factors DUOXA1 and DUOXA2, a necessary step for the maturation and activation of DUOX enzyme complexes. These proteins are co-localized with thyroperoxidase (TPO) in the apical membrane of thyrocyte and play a fundamental role in the synthesis of the thyroid hormones. TPO reduces the pairs of iodinated tyrosine in thyroglobulin in order to form thyroxine and triiodothyronine using hydrogen peroxide, heme beta and Ca2+ as cofactors (43, 44).

Glutathione peroxidase 3 (GPX3) catalyzes the reduction of H2O2 and is highly abundant in the thyroid gland (45). Therefore, excess of H2O2 and other reactive oxygen species (ROS) generated by DUOX system may be catalyzed by GPX3, protecting the cells against the oxidative damage. This is a finely regulated process between the balance among ROS generation and removal. Besides GPX3, thyrocytes express other members of the enzymatic antioxidant system, such as catalase, superoxide dismutases 1, 2 and 3 (with higher levels of SOD1), glutathione-disulfide reductase (GSR), several members of glutathione-S-transferase family (with higher levels of GSTP1 and GSTK1, respectively), several members of peroxiredoxin family (with higher levels of PRDX1, PRDX5 and PRDX3, respectively), thioredoxin (TXN) and thioredoxin reductases 1, 2 and 3 (with higher levels of TXNRD1) (45).

In thyroid dysfunctions, oxidative stress accompanied by thyroid epithelial cell inflammation is frequently observed. Both autoimmune thyroid diseases, Hashimoto’s thyroiditis and Graves’ disease present increased ROS production and decreased antioxidant activity (46). This imbalance was similarly observed in thyroid cancer cells; furthermore, these cells seem to reprogram their metabolic activity to survive in conditions of high oxidative stress and with a compromised antioxidant system (47).

The TH production, *per se*, is a high source of ROS in the thyrocyte. Nevertheless, we must include the oxidative phosphorylation by mitochondria in this equation. The mitochondrial respiratory chain consists of a series of reactions where electron transfer occurs. Initially, dehydrogenases collect electrons from the catabolic pathways and channel them to universal electron acceptors (NAD+, NADP+, FAD). The dehydrogenases linked to NAD+ removes two hydrogen atoms from their substrates, one of which is transferred as a hydride ion (H−) and the other is released as a proton (H+). Reducing equivalents of NADH are passed through a series of Fe–S centers to ubiquinone. The latter transfers electrons to cytochrome b and then to c (cytochrome oxidase), which accumulates electrons and then passes them to O2, reducing it to H2O. This process generates ROS that are inactivated by an enzymatic antioxidant complex (48). The metabolic homeostasis is maintained by balancing these steps according to the cellular need for ATP (49).

**Figure 2:**
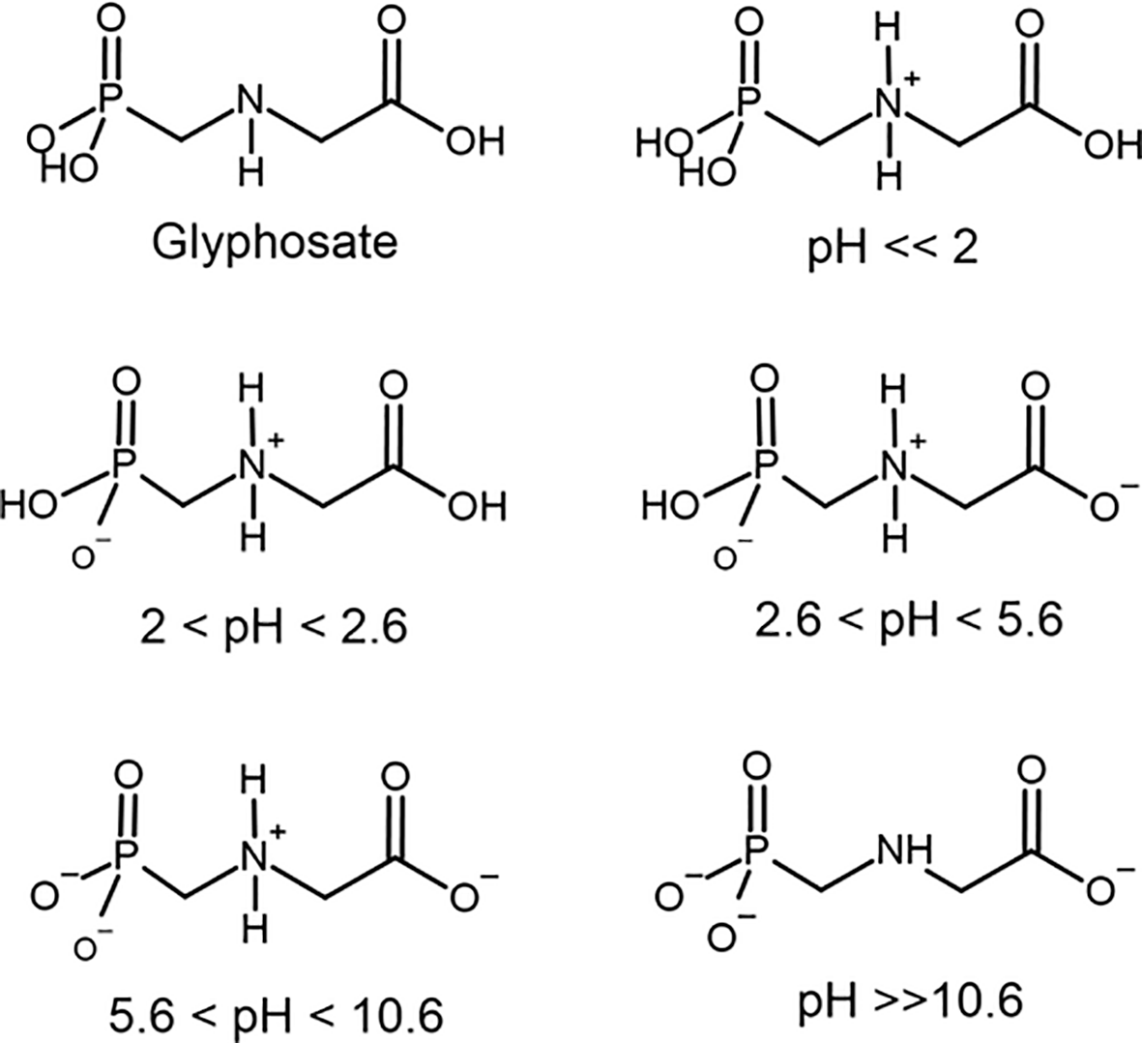
Chemical structure of glyphosate and its ionic forms under different pH conditions.

Therefore, it is possible to realize that the thyroid gland is very susceptible to changes in the balance between the generation and removal of ROS originated from both the synthesis of thyroid hormones and the oxidative phosphorylation. Several chemical compounds are associated with the disruption of the enzymatic antioxidant system (50, 51). In this context, our focus will be in glyphosate. Glyphosate is a phosphonomethyl derivative of the amino acid glycine. Glyphosate exists as a zwitterion in water (52) and is stable to hydrolysis at pH 5, 7, and 9 at 5 to 35°C (53). Zwitterions are polyelectrolytes that contain both positively and negatively charged groups, but the overall charge is neutral (54). Glyphosate comprises one basic secondary amino function and three ionizable acidic sites with dissociation constants (pKa) = 2.0 (phosphonic group), 2.6 (carboxylic group), 5.6 (ionization of the second O–H bond in phosphonic group) and 10.6 (protonate amine group) (**Figure 2**) (55–57). The amino, carboxylate and phosphonate groups in glyphosate coordinate strongly to metal ions (mainly transition metal) at near neutral pH where both the carboxylate and phosphonate will be deprotonated (58). Therefore, glyphosate can coordinate to metals as a unidentate and most frequently bidentate and tridentate ligand. Even tetradentate complexes are possible if both O–H bonds in phosphonate are ionized. Many of these structures are reported to have extensive hydrogen bonding stabilizing interactions (59). Stable metallic complexes with divalent (Cu, Zn, Mn, Ni, Cd, Pb, Ca and Mg, Ni), trivalent (Cr, Fe, Co, Al), monovalent ions (Li, Na, Ag) as well ammonium have been prepared (56, 59). The ability to bind anions or cations is influenced by the pH of the solution. At a pH below 2.0 glyphosate has a net positive charge due to protonation of the amino group, which contributes to an electrostatic attraction of negative charge counterions. But as the pH rises, the number of negative charges raise due to phosphonate and carboxylate groups ionization (**Figure 2**) Between pH 2.0 and 5.6, the phosphate acid is deprotonated, resulting in a negative charge allowing the interaction with monovalent cations. Between pH 5.6 and 10.6, the carboxylic acid is also deprotonated, further increasing the negative charge and increasing the possibility to bind to divalent cations. Above pH 10.6, all functional groups are deprotonated, and the negative charge is maximal, binding to trivalent cations. It is important to keep these different ionization forms of glyphosate depending on pH in mind because of their possible interaction with physiological process.

At gastric acid pH, the zwitterionic structure of glyphosate binds to anions and it is possible to hypothesize that iodide (I-) may interact with the agrochemical. Iodine and iodate from nutrition will be reduced in the gastrointestinal tract to iodide to be absorbed in the small intestine by sodium-iodide symporter (NIS) (60). Iodide glyphosate may not be transported by NIS, increasing its fecal excretion, and reducing its serum levels. At neutral and basic pH, the molecule develops a high capacity to form complexes with cations. Thus, from the pH of the stomach, intestine and internal environment, glyphosate may eventually chelate important elements such as iodide, calcium, magnesium, selenium, iron, zinc, etc., as observed for plants (61, 62). Glyphosate has a strong ability to form complexes with cationic macro and micronutrients in solution, affecting its absorption and the metabolism of the plants (61, 63).

Furthermore, glyphosate also uncouples oxidation from phosphorylation in the metabolic cycle by increasing the permeability of mitochondrial membrane to protons and Ca2+, disrupting the ATP synthesis (64). For example, in Sertoli cells, glyphosate induces oxidative stress with depletion of antioxidant defenses, culminating in necrotic cell death *via* Ca2+ dependent mechanism (opening voltage-dependent calcium channels (L-VDCC) and endoplasmic reticulum receptors (such as IP3 and ryanodine) (65).

## Glyphosate Toxicity in HPT Axis

The wide possibility of formulating pesticides containing glyphosate (glyphosate-based herbicides, GBH) gives a high complexity of toxicological analysis from an experimental point of view, but not regulatory. Because the registration of commercial products is based on the active compound, in this case only the glyphosate, with no tests that consider the combined effects of the commercial formulations proposed by the manufacturers. Thus, additive, synergistic or potentially toxic effects are not evaluated. Although GBH has been increasingly used since 1996, it was only in 2019 that the toxicological profile of glyphosate was published, with the release for public consultation of varied information that until then was not available (7).

There is greater toxicity in commercial glyphosate formulations than when using only glyphosate, indicating that the toxicity of the inert ingredients is greater than that of the active ingredient, and that their presence produces a greater toxic effect (66). Since human beings and the environment are exposed to GBH, not only to glyphosate, studies based on the real pesticide formulation are extremely relevant for raising awareness among regulatory agencies regarding the release and inspection of the use of these compounds and monitoring the occurrence of diseases in the exposed population.

In this regard, the identification of residues of glyphosate in soil, water and urine samples of human beings living or not near to crop farms raises the concern of International Agency for Research on Cancer (IARC/WHO) to support scientific evidences for potential glyphosate carcinogenicity and classified it as “probably carcinogenic to humans” (Group 2A) (9, 18–21). There are evidences between glyphosate exposure and the incidence of Non-Hodgkin lymphoma (15–17). Nonetheless, regulatory evaluations performed by EFSA and the ATSDR concluded that glyphosate is not toxic for the endocrine system or carcinogenic for human (7, 14, 67).

In a recent review, Agostini et al. (68) brought together studies related to the exposure of human cells to glyphosate showing that the effects can be different depending on the type of cell, including genotoxicity, hemolysis, apoptosis, abnormal antioxidant defense, growth abnormal cell, among others. Although endocrine changes are not as discussed as the other toxic effects, they are chronic diseases that produce deleterious effects throughout the individual’s life and require permanent attention by the health care system (69).

It is known that some chemicals which alter the genes involved in the HPT axis homeostasis may be associated with an increased incidence of thyroid dysfunction, such as hypothyroidism, hyperthyroidism and cancer (69–72). Among these, halogenated phenols, tributyltin, polychlorinated biphenyls and surfactants act on the hypothalamus and pituitary, while perchlorate, nitrates, thiocyanates, parabens and pesticides act on the thyroid gland itself (73, 74). Several other chemicals, such as phthalates, bisphenol A (BPA), brominated and perfluorinated flame retardants (PBDEs), polycyclic aromatic hydrocarbons (PAHs), perfluorooctane sulfonate (PFOS) and perfluorooctanoate (PFOA) were also reported to affect HPT axis (74–77). Specifically, organochlorine pesticides, polychlorinated biphenyls, aluminum, mercury, and vanadium could lead to thyroid autoimmunity (78). BPA and di-(2-ethylhexyl) phthalate (DEHP) would be related to the occurrence of Hashimoto’s thyroiditis and thyroid cancer (79). Increased risk of papillary thyroid carcinoma was also observed after the human exposure to polybrominated diphenyl ethers (80), flame retardants [BDE-209 and tris (2-chloro-ethyl) phosphate (TCEP)] (81) and cadmium (82).

Despite the knowledge of the deleterious effects of various chemicals on the thyroid gland, data on glyphosate toxicity is still scarce. The first evidence of thyroid toxicity was observed in amphibians (83). They are considered environmental sentinels for human health due to their sensitivity to the action of thyroid hormones during metamorphosis, since this process depends on the action of these hormones (84–86). Tadpoles were exposed to GBH formulations containing or not polyoxyethyleneamine (POEA) surfactant, glyphosate or only POEA surfactant and changes in metamorphosis were observed in tadpoles exposed to GBH containing POEA surfactant and in the group exposed only to POEA, but not in the group treated with glyphosate and GBH without POEA. In conclusion, POEA is toxic for HPT axis, but not glyphosate (83). As mentioned, the regulatory agencies only evaluated the glyphosate, not its formulation. Therefore, this study was a strong scientific support for the safety of glyphosate for the endocrine system.

Nine years passed before the next study questioning the thyroid toxicity of glyphosate in amphibians was published (**Table 1**). Lanctot et al. (87) observed a decrease in Trhb expression in the brain of tadpoles treated with GBH. Navarro-Martín et al. (88) observed an increase in Dio2 and Dio3 expression in the brain and a delay in metamorphosis of tadpoles treated with GBH. Thereafter, other studies reporting deleterious effects of glyphosate formulations in the metamorphosis were conducted (89–95).

**Table 1:** Summary of the thyroid toxic effects after glyphosate or glyphosate-based herbicides (GBH) exposure in amphibians.

Chemical	LOAEL for the study	Experimental model	Age at beginning of exposure	Exposure duration	Exposure route	Main results	Reference
GBH (containing or not POEA), Glyphosate, or POEA surfactant	6 mg/L	Tadpoles	Gosner stages (GS) 20 and 25	24 and 96 h	Water (aquaria)	In GBH containing POEA and POEA: ↑ Tail damage ↓ Tail length ↑ expression Thrb in tail	Howe et al. (83)
GBH (Roundup WeatherMax and Vision, Monsanto Co.)	0.21 mg/L	Tadpoles	GS 25	two pulses (4 consecutive days) within 2 weeks	Water (aquaria)	↓ expression Thrb in brain	Lanctôt et al. (87)
GBH (VisionMax, Monsanto Co.)	0.21 mg/L	Tadpoles	GS 25	41 days	Water (aquaria)	↑ expression Dio2 and Dio3 in brain Delayed metamorphosis	Navarro-Martín et al. (88)
GBH IPA (Aquamaster, Monsanto Co.), or GHB IPA+ Agri-dex surfactant, or GHB IPA+ Competitor surfactant	GHB IPA+ Competitor surfactant: 711 mg/L	Tadpoles	GS 35 to 38	24 and 48h	Water (aquaria)	Both herbicide mixes were more toxic than the active ingredient alone ↑Tadpole mortality in the herbicide mixture	Vincent and Davidson (89)
GBH (Roundup ULTRA MAX^®^, Monsanto Co.) or technical-grade glyphosate	GHB: 0.0007 mg/L GLY: 15 mg/L	Tadpoles	GS 25 and 36	96 h	Water (aquaria)	GBH Swimming activity was affected Effects on growth, development, and abnormalities GLY Effects on growth, development, and abnormalities	Bach et al. (90)
GBH (Roundup Original^®^, Monsanto)	18 μgl^−1^	Tadpoles	GS 25	7 days	Water (aquaria)	↓ Glycogen ↑ Total lipid levels ↓ Triglyceride levels ↑ Cholesterol levels ↓ Total protein ↑ LPO ↓ Glycogen, cholesterol, total protein and triglyceride levels in the liver ↓ Glycogen, Total lipid, Triglyceride in the tissue muscle ↑Total protein in the muscle ↑ Lipid peroxidation levels in the Gills, liver and tissue muscle ↑Weight and size	Dornelles and Oliveira (91)
GBH (Glyphogan^®^Classic, Monsanto Co.)	2 mg a.e/L	Tadpoles	GS 25	1st, 2nd, 3rd, 4th, or 5th period of their larval development, each period lasting 9 days. Or even throughout the experiment period (5th period larval)	Water (aquaria)	↓ Body mass at metamorphosis during the entire experiment ↑ Time to metamorphosis during the entire experiment Slow development of tadpoles exposed throughout the experimental period Low dose individuals in the first exposure period group developed slower High dose ↓ Body mass in the 1st, 4th, and 5th period Individuals in the 1st,2nd, 3rd, 4th exposure period group developed slower	Mikó et al. (92)
GBH (Roundup1UltraMax and Focus Ultra, Monsanto Co.)	13.5 mg/L	Tadpoles	GS 25	96 h	Water (aquaria)	Delayed metamorphosis	Wagner et al. (93)
GBH (Roundup original^®^DI)	144 µg/L	Tadpoles	GS 25–26	acute assay: 96 h chronic assay: 14 days	Water (aquaria)	Tadpoles showed shorter lengths and lower masses ↑ Malformation in the mouth, the epithelium color and bowel edema	Herek et al. (94)
GBH (Roundup^®^ Star) or Glyphosate (PESTANAL^®^, analytic grade, 45521, Sigma-Aldrich)	–Stage 8: 37.8 mg active ingredient L^−1^ for embryos –Stage 46: 45.1 mg active ingredient L^−1^ for tadpoles	Embryos and tadpoles	–FETAX test: stage 8 embryos –Tadpole-toxicity bioassays: stage 46	24, 48, 72 and 96 h	Water/in a standard Frog Embryo Teratogenesis Assay Xenopus (FETAX) test medium	GBH: Significant inhibition of embryonic growth ↓Activity of GR, CaE, AChE, and SOD	Turhan et al. (95)

It has been noticed that in recent years the concern about the toxicity of the commercial formulation of glyphosate has increased. From 2017, some studies in rats and mice suggest that GBH is toxic to the HPT axis (**Table 2**), reinforcing what had already been observed for amphibians. de Souza et al. (96) evaluated the regulation of TSH production in adult male offspring of Wistar rats perinatally exposed to GBH (5 or 50 mg/kg) during the period of hypothalamic sexual differentiation (gestational 18 to postnatal day 5). Decreased TSH serum was observed along with a reduction of deiodinases expression in the hypothalamus, increased expression of thyroid hormone receptors in the pituitary and liver, and a metabolic profile compatible with that observed in animals with hypothyroidism. Manservisi et al. (97) administered glyphosate or GBH at 1.75 mg/kg bw/day in drinking water from gestational day 6 until 120 days old. Male and female offspring were sacrificed at PND73 (6 weeks) or PND125 (13 weeks) and an increase in serum TSH levels in males was observed after 6 weeks of treatment for glyphosate, and after 13 weeks for GBH. Hamdaoui et al. (98) evaluated oral exposure to 126 mg/kg GBH during 60 days in female Wistar rats and investigated its action on bone tissue in correlation with the hormonal status of estrogen and thyroid gland. Histological and immunohistochemical analysis of the thyroid showed that after the exposure there was a relative increase in the appearance of follicles at rest with a consequent decrease in the number of active follicles, in addition to the appearance of macrophages, dilated follicles and flattened epithelium, with decreased volume of colloid. These findings correlate with a state of hypothyroidism. The study also showed that hormone levels of T3 and free T4 decreased in the treated animals, while TSH levels increased and estrogen levels decreased, leading to an osteoporosis condition. Adult female Kunming Mice exposed to 250 mg/kg GBH during 7 days by gavage presented increased levels of serum TRH, with decreased TSH, T3 and T4. The gene expression of Dio 2 and Mct8 were decreased in the hypothalamus, while in the pituitary the expression of Dio2, Mct8 e Trhr were increased. In the thyroid, the expression of Nis, Tpo, Tg and Tshr were decreased. All levels of the HPT axis were deregulated (99).

**Table 2:** Summary of the thyroid toxic effects after glyphosate or glyphosate-based herbicides (GBH) exposure in rats and mice.

Chemical	LOAEL for the study	Experimental model	Age at beginning of exposure	Exposure duration	Exposure route	Main results	Reference
GBH (Roundup Transorb, Monsanto Co.)	5 mg/kg	♂ Wistar rats (adult male offspring from dams treated with GBH)	Gestational day 18 to postnatal day 5	10 days	Gavage to the mothers	↓TSH Altered expression of several genes related to thyroid function Metabolomics changes similar to hypothyroidism	de Souza et al. (96)
GBH (Roundup Bioflow, Monsanto Co.) and glyphosate	1.75 mg/kg	♂♀ Sprague–Dawley rats	Gestational day 6 to postnatal day 120	6 weeks or 13 weeks	Drinking water	↑ TSH ♂ (glyphosate—6 weeks) ↑ TSH ♂ (GBH—13 weeks)	Manservisi et al. (97)
GBH (Kalach 360 SL, Monsanto Co.)	126 mg/kg	♀ Wistar rats	Not informed	60 days	Gavage	↑ TSH ↓FT3 and FT4 ↓colloid volume in thyroid ↓ calcitonin	Hamdaoui et al. (98)
GBH (brand not mentioned)	250 mg/kg	♀ Kunming Mice	Four-week-old	7 days	Intragastric	↑ TRH serum ↓ TSH, T3 and T4 serum ↓ Relative expression of Dio2 and Mct8 in the hypothalamus ↑ Relative expression of Dio2, Mct8 and Trhr in the pituitary ↓ Relative expression of Nis, Tpo, Tg and Tshr in thyroid tissue	Zhang et al. (99)

In humans, three studies evaluated occupational exposure to pesticides and suggested that GBH might be involved in thyroid dysfunction (**Table 3**). Kongtip et al. (13) observed higher levels of TSH, FT3, T3, and T4 in conventional farmers compared to organic farmers, while glyphosate was the most used compound, followed by paraquat and 2,4-dichlorophenoxyacetic acid (2,4-D). In Brazil, researchers evaluated the levels of T3, T4 and TSH in soy producers who used pesticides, including glyphosate, and found a significant decrease in TSH and an increase in T3 and T4 (100). Another study evaluated the association between the use of pesticides, including glyphosate, and the incidence of hypothyroidism in applicators over a period of 20 years and found that the risk of hypothyroidism was significantly increased due to contact with these substances (12). It is noteworthy that in these studies the evaluated population was not exclusively exposed to GBH, and detectable levels of other pesticides were also found. This does not exclude the probable toxicity caused by GBH, but also does not make it possible to attribute it only to him. This is a characteristic of studies involving human beings and makes their interpretation fragile. Thus, more studies are needed to reinforce this hypothesis of thyroid toxicity in humans and that regulatory actions can be applied to GBH.

**Table 3:** Summary of the thyroid toxic effects after glyphosate-based herbicides (GBH) exposure in human.

Gender	Age at beginning of exposure	Exposure duration	Exposure route	Main results	Reference
♀♂	50–53 years old	February–December 2016	Occupational exposure	↑ Levels of T4	Kongtip et al. (13)
♂	43.9 ± 11.5 years old	The study was conducted during the high exposure period to pesticides	Occupational exposure	↓ TSH ↑ T3 and T4	Bernieri et al. (100)
♀♂	≤45 years to 65	1999–2016	Occupational exposure	Elevated risk of hypothyroidism	Shrestha et al. (12)

It is also clear throughout this review that the toxicity of GBHs is significant and that there is a need to consider the other ingredients of the commercial formulation in toxicological evaluations. Surfactants, additives and other substances are used in different proportions to obtain the final commercial formulation. This would practically make it impossible to use the toxicology procedures by regulatory agencies as they are done today, because it would require an extremely high amount of laboratory animals and the other procedures associated with these analyzes. However, other methods should be encouraged, such as an initial screening of these mixtures by *in vitro* and *in silico* methodologies.

## Future Perspectives

Finally, in addition to the known mechanisms of regulation involving the hypothalamic-pituitary-thyroid axis widely presented in this review, recent studies indicate that the intestine has an important role in the control of thyroid function, forming the Thyroid–Gut-Axis. According to this proposal, the intestinal microbiota affects mechanisms related to the absorption of iodide, conversion of T4 to T3, and also participates in the modulation of the immune system (11, 101). This is corroborated by studies where gut microbiota dysbiosis was correlated to Hashimoto’s thyroiditis (102–104), Graves’ disease (104), thyroid cancer and thyroid nodules (105). In parallel in the field of toxicology, recent studies indicate that GBH is able to affect the intestinal microbiome of mice (106), rats (107–112), honeybees (113, 114), Japanese quails (115) and *Daphnia magna* (116). A possible explanation for this toxicity is that some classes of bacteria require the shikimate pathway (5-enolpyruvylshikimate-3-phosphate synthase, EPSPS) for their metabolism. The glyphosate mechanism of action is through inhibition of the growth of weeds by interfering with the production of essential aromatic amino acids by inhibiting EPSPS, which is responsible for chorismate biosynthesis, an intermediate in the biosynthesis of phenylalanine, tyrosine and tryptophan (7). Then, from the moment glyphosate inhibits it, these bacteria are unable to maintain normal levels in the gut microbiome (111, 113–115). Thus, new studies evaluating the involvement of the gut-thyroid axis are relevant in advancing the knowledge of glyphosate toxicity on thyroid function.

## Final Considerations

The toxicity of glyphosate on thyroid function and its participation in the increasing incidence of thyroid diseases is still a very controversial subject. The main points of this review were:

- The regulation of the HPT axis is very complex, with several steps that can be affected by xenobiotics;
- The HPT axis has the capacity to adapt to different stress situations (allostasis), confusing the interpretation of EDC effects;
- The synthesis of THs depends on the formation of H_2_O_2_, and the ROS formed are neutralized mainly by selenoproteins;
- Glyphosate has a high probability of binding to anions and cations in pH compatible with those observed in body compartments; the real occurrence/extension/consequences of this effect should be evaluated experimentally;
- Glyphosate alters the absorption of selenium, the functioning of the Ca^++^ channels and disrupts oxidative phosphorylation, thus it can affect the thyroid function;
- Oxidative stress is present in autoimmune thyroiditis and thyroid carcinomas;
- The HPT axis is recognized as the target of EDCs;
- Commercial formulations (GBH) are more toxic than glyphosate;
- Few thyroid toxicology studies have been performed to date;
- In tests validated by regulatory agencies, the thyroid changes presented are disregarded, since allostatic regulation is observed;
- Glyphosate is detected in the urine of residents of rural and urban environments;
- There is a correlation between farmers’ exposure to GBHs and altered thyroid hormone levels or incidence of thyroid pathologies;

That presented, two conclusions appear to be more important: (1) there are few studies evaluating the mechanisms of toxicity (where, how and if it occurs), which makes the extrapolation of correlations between human exposure and toxicity somehow fragile; (2) It might be time to start considering testing the toxicity of commercial formulations, as these represent the actual human and environmental exposure.

## Author Contributions

RM and MR contributed to conception and design of the review. IO and PB-S wrote the section The Maintenance of Hypothalamus–Pituitary–Thyroid Axis Homeostasis and Its Allostasis-Adaptive Responses. RM, MR, and YT wrote the section Thyroid Hormones Synthesis Pathway as a Possible Target to Toxicants. VD, RM, and MR wrote the section Glyphosate Toxicity in HPT Axis. JD, RM, and MR wrote the section Future Perspectives. RM, MR, and AM wrote the sections Introduction and Final Considerations. All authors contributed to the article and approved the submitted version.

## Funding

JMO was recipient of scholarship instead (Coordenação de Aperfeiçoamento de Pessoal de Nivel Superior—Brasil [CAPES]—Finance Code 001). RMR is recipient of Conselho Nacional de Desenvolvimento Científico e Tecnológico- BRASIL productivity grant (CNPq 09/2020, # 309256/2020-8)

## Conflict of Interest

The authors declare that the research was conducted in the absence of any commercial or financial relationships that could be construed as a potential conflict of interest.

## Abbreviation

2,4-D, 2,4-dichlorophenoxyacetic acid; AA, acrylamide; ATSDR, Agency for Toxic Substances and Disease Registry; BPA, bisphenol A; DEHP, di- (2-ethylhexyl) phthalate; DIO1, deiodinase 1; DIO2, deiodinase 2; DIO3, deiodinase 3; DUOX1, dual oxidase 1; DUOX2, dual oxidase 2; DUOXA1, dual oxidase maturation factor 1; DUOXA2, dual oxidase maturation factor 2; EFSA, European Food Safety Authority; GBH, glyphosate-based herbicides; GPX3, Glutathione peroxidase 3; GS, Gosner stages; GSR, glutathione-disulfide reductase; GSTK1, glutathione S-transferase kappa 1; GSTP1, glutathione-S-transferase pi 1; H2O2, hydrogen peroxide; HPT, hypothalamus–pituitary–thyroid axis; IARC, International Agency for Research on Cancer; LOAEL, lowest-observed-adverse-effect level; L-VDCC, voltage-dependent calcium channels; MCT8, Monocarboxylate Transporters 8; MCT10, Monocarboxylate Transporters 10; NIS, sodium-iodide symporter; NTIS, non-thyroidal illness syndrome; OATP1C1, Solute Carrier Organic Anion Transporter family member 1C1; PAH, polycyclic aromatic hydrocarbons; PBDE, Polybrominated diphenyl ethers; PFOA, perfluorooctanoate; PFOS, perfluorooctane sulfonate; POEA, polyoxyethyleneamine; PRDX1, peroxiredoxin 1; PRDX3, peroxiredoxin 3; PRDX5, peroxiredoxin 5; PRL, Prolactin; PVN, hypothalamic paraventricular nucleus; RGD, arginyl-glycyl-aspartic acid domain; ROS, reactive oxygen species; rT3, reverse T3; SOD1, superoxide dismutase 1; T4, 3,5,3’,5’-tetraiodothyronine or thyroxine; TACITUS, thyroid allostasis in critical illnesses, tumors, uremia, and starvation; TBG, Thyroid hormones binding proteins; Te, 3,5,3’-triiodothyronine or triiodothyronine; TH, thyroid hormones; THRA, Thyroid Hormone Receptors Alpha; THRB, Thyroid Hormone Receptors Beta; TPO, thyroperoxidase; TRE, thyroid hormone-responsive element; TRH, Thyrotrophin-Releasing Hormone; TRH-R, TRH receptor; TSH, Thyroid-Stimulating hormone; TSH-R, TSH receptor; TXN, thioredoxin; TXNRD1, thioredoxin reductases 1.
